# Recent advances in therapies for primary myelofibrosis

**DOI:** 10.12703/r/12-23

**Published:** 2023-09-26

**Authors:** William Vainchenker, Nasrine Yahmi, Violaine Havelange, Caroline Marty, Isabelle Plo, Stefan N Constantinescu

**Affiliations:** 1 INSERM, UMR1287, Gustave Roussy, Villejuif, France; 2 Université Paris-Saclay, UMR1287, Gustave Roussy, Villejuif, France; 3 Gustave Roussy, UMR1287, Villejuif, France; 4 de Duve Institute, Université catholique de Louvain, Brussels, Belgium; 5 Cliniques universitaires Saint Luc, Department of Hematology, Université Catholique de Louvain, Brussels, Belgium; 6 Ludwig Institute for Cancer Research, Brussels, Belgium; 7 WEL Research Institute, WELBIO Department, Wavre, Belgium; 8 Ludwig Institute for Cancer Research, Nuffield Department of Medicine, Oxford University, Oxford, United Kingdom; 9

**Keywords:** Myeloproliferative neoplasms, myelofibrosis, therapeutic approaches

## Abstract

Primary myelofibrosis (PMF), polycythemia vera (PV) and essential thrombocythemia (ET) form the classical *BCR-ABL1*-negative myeloproliferative neoplasms (MPNs) that are driven by a constitutive activation of JAK2 signaling. PMF as well as secondary MF (post-ET and post-PV MF) are the most aggressive MPNs. Presently, there is no curative treatment, except allogenic hematopoietic stem cell transplantation. JAK inhibitors, essentially ruxolitinib, are the therapy of reference for intermediate and high-risk MF. However, presently the current JAK inhibitors behave mainly as anti-inflammatory drugs, improving general symptoms and spleen size without major impact on disease progression. A better understanding of the genetics of MF, the biology of its leukemic stem cells (LSCs), the mechanisms of fibrosis and of cytopenia and the role of inflammatory cytokines has led to new approaches with the development of numerous therapeutic agents that target epigenetic regulation, telomerase, apoptosis, cell cycle, cytokines and signaling. Furthermore, the use of a new less toxic form of interferon-α has been revived, as it is presently one of the only molecules that targets the mutated clone. These new approaches have different aims: (a) to provide alternative therapy to JAK inhibition; (b) to correct cytopenia; and (c) to inhibit fibrosis development. However, the main important goal is to find new disease modifier treatments, which will profoundly modify the progression of the disease without major toxicity. Presently the most promising approaches consist of the inhibition of telomerase and the combination of JAK2 inhibitors (ruxolitinib) with either a BCL2/BCL-xL or BET inhibitor. Yet, the most straightforward future approaches can be considered to be the development of and/or selective inhibition of JAK2V617F and the targeting MPL and calreticulin mutants by immunotherapy. It can be expected that the therapy of MF will be significantly improved in the coming years.

## Introduction

The classical *BCR-ABL1*-negative myeloproliferative neoplasms (MPNs) (denoted here thereafter MPNs) include three different disorders, essential thrombocythemia (ET), polycythemia vera (PV) and primary myelofibrosis (PMF). They may be seen as 3 stages of the same disorder as they are all driven by constitutive activation of JAK2 by mutations in 3 genes^1,2^. Disease phenotype and prognosis are related to the precise disease driver mutation along with its genetic status (heterozygous or homozygous), but also to the presence of other acquired “clonal” driver mutations. The latter are also associated with other myeloid malignancies and clonal hematopoiesis, but they do not *per se* induce MPN. The combination of acquired mutations, their number and order of acquisition play a central role in the phenotype and prognosis^1–3^. In addition, inherited genetic factors and environmental cues such as inflammation and iron metabolism dysregulation may also impact phenotype and prognosis^1^.

PMF is the most severe MPN. Diagnosis is based on bone marrow histology with the presence of megakaryocyte (MK) proliferation and marrow fibrosis (grade 2–3) associated with osteosclerosis in advanced cases^4,5^. Secondary MF is preceded by an ET or a PV. An overt PMF can be preceded by an early-PMF (pre-fibrosis) that shares many features with ET, but differs by the presence of a splenomegaly, increased bone marrow cellularity with dysplastic MK proliferation and eventually low-grade marrow fibrosis (grade<2). Most PMFs have disease driver mutations, except a subgroup denoted as triple negative PMF^5,6^.

The severity of the disease is related to the risk of leukemic transformation (14–25%), severe cytopenia, thromboembolic, hemorrhagic and infectious complications, cardiovascular disorders and cachexia.

There is no curative treatment of MF except allogeneic hematopoietic stem cell (HSC) transplantation, which remains risky, despite major progress in its management. Thus, to progress in the therapy of PMF, it is crucial to better understand the precise molecular bases of MPN and MF development.

## MF and mutations

### Disease driver mutations

MPN MF can be induced by 3 disease driver mutations. *JAK2*V617F is the predominant mutation present in around 55%–60% PMF^1,2^. *JAK2*V617F is a gain of function (GOF) mutation not located in the kinase domain, but in the pseudo-kinase (PK) domain. The V617F mutation activates the kinase domain by dimerization of the mutated PK domains, stabilizing a dimer state and removing negative regulation on the kinase domain^7^. The mutation can be heterozygous, or homozygous. JAK2V617F can induce constitutive signaling downstream of the three main “myeloid” homodimeric receptors (EPOR, G-CSFR, MPL), explaining that it may induce the three diseases. The 3 main signaling pathways include the STAT activation (STAT1, STAT3 and STAT5, according to the receptors), the PI3K/AKT/mTOR and the RAS/MAPK pathways. JAK2V617F not only induces proliferation of hematopoietic stem cell progenitor (HSCP) cells but gives a strong proliferative advantage to maturing precursors. It also activates or primes mature cells, inducing an inflammatory response, mainly through STAT3 activation and favoring thrombosis. Phylogenic reconstitution of the clone history in *JAK2*V617F MPN has shown that the mutation arises decades before disease development or *in utero,* giving the possibility of an early therapeutic approach^8^.

*CALR* mutations (mut) are the predominant disease driver mutation in *JAK2 and MPL* wild type (WT) PMF^1,2^. All described mutations are deletions/insertions in the exon 9, inducing a frameshift (-1/+2), creating a new C-terminus with positively charged and hydrophobic residues and lacking the KDEL endoplasmic reticulum (ER) retention signal. The two most frequent mutations are del52 (type-1) and ins5 (type-2), del52 mutations being enriched in PMF in comparison to ET^9^. CALR is a chaperone of the ER that plays a major role in the quality control of secreted glycoproteins and in calcium metabolism. The CALRmut, thanks to their C-terminus, specifically bind to N-glycosylated MPL via the lectin domain^10–12^. The CALRmut/MPL complex traffics to the cell surface; CALRmut are oligomers that dimerize MPL leading to persistent JAK2 activation^13,14^. CALRmut induce cell signaling at the cell surface via MPL or by being secreted and behaving as rogue cytokines that secondary bind to the immature sugars of MPL only present in the clone and by oligomerizing with endogenous CALRmut^15^. Thus, CALRmut can be directly targeted by different approaches. CALRmut may also exert effects via impaired calcium ER retention and subsequent activation of an ER stress response^16,17^. While *CALR*mut MPN develop on average nearly a decade before *JAK2*V617F MPN, *CALR*mut are acquired later in adult life, as inferred by mathematical modeling^18^. However, in a case of monozygotic twins it was shown that the mutation was acquired during fetal life^19^.

GOF *MPL* mutations are much rarer (around 5%). Mutations occur either in the cytosolic juxta-membrane domain at W515, usually W515L/K, or in the transmembrane domain, *MPL*S505N, both types leading to MPL dimerization and TPO independent megakaryopoiesis^1,2,20^. Other mutations can be found all along the *MPL* sequence. They are much rarer and exert weak GOF^20^.

### “Clonal” driver mutations

In 80% of PMF, other “clonal” driver mutations are found that impact disease and prognosis. *ASXL1*, *EZH2*, *SRSF2* and *IDH1/IDH2* mutations are associated with an adverse prognosis^5^. Other mutations such as in *N-RAS*, *K-RAS, CBL* and other splicing genes induce also a worsened prognosis^5^. The role of these mutations in prognosis may also depend on the disease driver mutations. For example, the prognosis of *CALR* type-1 mutations is not altered by an *ASXL1* mutation.

The role of *TP53* mutations in prognosis is controversial but may be important in the choice of therapy^21^.

PMF is a heterogeneous disease concerning its prognosis due to the complex molecular alterations. Scores have been developed to stratify the prognosis and improve the choice of therapy based on clinical data, very high risk cytogenetic abnormalities (-7, i(17q), inv(3)/3q21, 12p-/12p11.2, 11q-/11q23, or other autosomal trisomies not including +8/+9) and high-molecular-risk mutations (HMR) (*ASXL1*, *SRSF2*, *EZH2*, *IDH1* and *U2AF1*Q157)^4,5^.

## Physiopathology of the marrow fibrosis

Marrow fibrosis (myelofibrosis) *per se* is characterized by the accumulation of extracellular matrix (ECM) fibers, namely collagen, that disorganizes the bone marrow environment and impairs hematopoiesis. It starts with the formation of a loose network of reticular fibers made of collagen III (grade-1) and then progresses with the accumulation of fibers composed of collagen I (grade-2) that will further accumulate with the appearance of an osteosclerosis (grade-3)^6^.

Marrow fibrosis can be associated with very different pathologies, such as auto-immune disorders, cancer metastases, lymphoma and myeloid malignancies. PMF may progress in two stages: one with a major myeloproliferation and the second called “myelodepletive” characterized by cytopenia^22^. PMF is associated with important extra-medullary hematopoiesis (EMH), more particularly in the spleen. The marrow fibrosis is usually considered to be reactive, as consequence of a cross-talk between the clonal disorder and stromal cells, which secrete ECM. ECM is also regulated by MKs that synthesize different types of collagen and extra-domain-A fibronectin and lysyl-oxidase, an enzyme playing an important role in ECM organization^23,24^.

TGF-β1 plays a central role in the development of all types of fibrosis and appears indispensable for marrow fibrosis development in mouse models^25,26^. It acts on mesenchymal stem cells (MSC) by inducing their proliferation and programming them to myofibroblasts and osteoblasts. TGF-β1 is secreted as an inactive latent form in a complex with TGF-β propeptide also called latency-associated protein (LAP), and latent TGF-β binding protein (LTBP). TGF-β1 is activated by different mechanisms including the interaction with αv integrins or the ECM such as thrombospondin 1 or the production of reactive oxygen species (ROS)^25^. In MF patients, the latent and active TGF-β1 levels are increased in bone marrow and plasma. The mechanism of TGF-β1 activation in MF is not clearly established and could be either related to the ROS level or the presentation of latent TGF-β1 on the cell surface by GARP through LAP binding to be subsequently activated by integrins. Initially it was found that GARP is only expressed on immune cells, but it is also present on the membrane of MKs/platelets as well as of some stromal cells^27,28^.

The other important determinant is the role of inflammation, more particularly of pro-fibrotic cytokines. The development of inflammation is an early event in the development of MPN. It has been shown that numerous plasma pro-inflammatory cytokines, such as IL8, IL12 and IL15 are predictive of the PMF prognosis^5^. The hematopoietic cells of the MPN as well as hematopoietic cells not belonging to the clone and non-hematopoietic cells are involved in inflammation^29^. It has been underscored that stromal cell inflammation may precede the development of the disease^30^. Pro-inflammatory cytokines may act by: (a) promoting a clonal advantage for cytokines (IL1β, IL8, TNFα and IL6)^31^; (b) inducing ROS release that may activate the latent TGF-β1; (c) reprogramming the Gli^+^ Lep^+^ MSC to myofibroblasts (IL1β and IL6 and alarmins S1008A/S1009A);^32^, and (d) acting on TGF-β1 synthesis and activation (IL13)^28^. Overall, the JAK/STAT3 and the NFκB pathways are important in the synthesis of pro-inflammatory cytokines and their effects^29,33^.

MKs appear to be a key cell determinant in marrow fibrosis development, as suggested by their close localization with collagen fibers^34^. They are the most important source of TGF-β1 in the bone marrow with a latent form that can be presented at the cell surface. In addition, MKs synthesize pro-inflammatory cytokines, chemokines and pro-angiogenic factors as well as ECM. The secretion of TGF-β1 and other pro-inflammatory cytokines, which are packaged in α-granules, is probably favored by the dysmegakaryopoiesis and the activation of MKs, as shown by the cell surface expression of P-selectin associated with an increased emperipolesis^35^.

These last years, the role of monocytes in marrow fibrosis development has been highlighted. Indeed, monocytes can release a lot of pro-inflammatory cytokines. In addition, SLAMF7 monocytes can differentiate to fibrocytes that are also considered as important mediators of marrow fibrosis in the *JAK2*V617F mouse model^36,37^. In this case, the marrow fibrosis will not only be a reactive process, but may directly derive from the clone. Recently, it has been suggested that Tregs play a central role in TGF-β1 production and activation and may also limit the CD8 T cell immune reaction against the clone^27^. Mast cells may also be involved in TGF-β1 activation by IL13 secretion^28^.

It must be underscored that an increased level of TPO in the mouse model called TPO^high^ or the administration of MPL agonist, such as romiplostim, in mouse and in non-human primates leads to the development of a severe marrow fibrosis^38^. Similarly, in humans, administration of romiplostim may induce a moderate marrow fibrosis, which regresses at the arrest of the treatment^39^. In addition, the *MPL*W515L retroviral mouse model and *MPL*S504N knock in (KI) mice (human *MPL*S505N) develop a rapid marrow fibrosis^40,41^. Moreover, the genetic reversion of *Jak2*V617F to *Jak2* WT in *Jak2*V617F KI mice induces a marrow fibrosis regression^42^. All these results demonstrate that a very strong activation of the TPO/MPL/JAK2 pathway is sufficient to induce the marrow fibrosis development and a pro-inflammatory state.

Presently, none of the pharmaceutical approaches have really improved the prognosis of high-risk MF^4,43–45^. Thus, besides JAK2 inhibitors and IFNα, many new therapies are in development, targeting different cellular processes, such as apoptosis, cell cycle, epigenetic regulators, signaling pathways, telomerase, or directly the leukemic stem cell (LSC). Many of these therapies are developed in association with JAK2 inhibitors with the risk of increasing hematological toxicity. The two principal goals of these new therapies are to: (a) significantly impact the clonal disorder and thus to revert the MF and to increase patient survival, and (b) provide alternative therapies in case of JAK2 inhibitor resistance or intolerance^4,43–45^. Other therapies have also been designed to improve the cytopenia and to target the marrow fibrosis. Selected ongoing clinical trials are shown in Table 1 A and B.

**Table 1.** Selected clinical trials. **A) Ongoing clinical trials; recruiting patients**

drug	mechanism of action	ongoing clinical trial recruiting	MF patients
**Pacritinib**	jak inhibitor	**phase 3** : Pacritinib 200mg BID vs P/C therapy (PACIFICIA)	≥ int1 risk platelets < 50,000/µL JAK inh naive (1^st^ L) limited exposure JAK inh (2^nd^ L)
Pegasys	interferon	phase 1/2 : Pegasys + ruxolitinib (RUXOPeg) phase2/3 : COMBI-I and COMBI-II	≥ int1 risk JAK inh naive (1^st^ L)
Rogepinterferon alpha 2b	interferon	phase 2	prePMF/low/int1 risk
**Imetelstat**	telomerase inhibitor	**phase 3** : Imetelstat vs BAT	int2/high risk R/R JAK inh (2^nd^ L)
**Pelabrasib**	BET inhibitor	**phase 3** : Pelabrasib vs placebo + ruxolitinib (MANIFEST-2)	≥ int1 risk JAK inh naive (1^st^ L)
BMS-986158	BET inhibitor	phase 1b/2 : monotherapy or + ruxolitinib or + fedratinib	≥ int1 risk JAK inh naive (1^st^ L)
ABBV-744	BET inhibitor	phase 1b : monotherapy or + navitoclax or + ruxolitinib	int2/high risk R/R /intolerant JAK inh (2^nd^ L)
Bromedemstat	LSD1 inhibitor	phase 2 : Bromedemstat + ruxolitinib	≥ int1 risk A : R/R/intolerant JAK inh (2^nd^ L) B : JAK inh naive (1^st^ L)
**Parsaclisib**	PI3Kẟ inhibitor	**phase 3** : Parsaclisib vs placebo + ruxolitinib (LIMBER-313)	≥ int1 risk JAK inh naive (1^st^ L)
AUY922	HSP90 inhibitor	phase 2	ineligible JAK inh (1^st^ L) R/R JAK inh (2^nd^ L)
TP-3654	PIM inhibitor	phase 1/2	≥ int1 risk R/R JAK inh (2^nd^ L) ineligible JAK inh (1^st^ L)
abemaciclib	CDK4/6 inhibitor	phase 1	≥ int1 risk inadequate resp JAK inh (2^nd^ L)
TL-895	BTK tyrosine kinase inhibitor	phase 2 Phase 1b/2 : TL-895 + ruxolitinib	≥ int1 risk R/R/intolerant JAK inh (2^nd^ L) ineligible JAK inh (1^st^ L) JAK inh naive (1^st^ L) suboptimal resp JAK inh (2^nd^ L)
**Navitoclax**	BCL2, BCL-xL,BCL-W inhibitor	**phase 3** : navitoclax + ruxolitinib vs BAT (TRANSFORM-2)	int2/high risk R/R /intolerant/suboptimal resp JAK inh (2^nd^ L)
**KRT-232**	MDM2 inhibitor	**phase 2/3** : KRT-232 vs BAT phase 1b/2 : KRT-232 + ruxolitinib	≥ int1 risk failure of JAK inh (2^nd^ L) ≥ int1 risk suboptimal resp JAK inh (2^nd^ L)
Selinexor	XPO1 inhibitor	phase 2 : selinexor vs P/C therapy phase 2 : selinexor single arm phase 1/2 : selinexor + ruxolitinib	≥ int1 risk R/R/intolerant JAK inh (2^nd^ L) ≥ int1 risk – JAK inh naive (1^st^ L)
GB2064	LOXL2 inhibitor	phase 2a	int2/high risk R/R/intolerant JAK inh (2^nd^ L) ineligible JAK inh (1^st^ L)
PXS-5505	LOX inhibitor	phase 1/2a	int2/high risk R/R/intolerant JAK inh (2^nd^ L) ineligible JAK inh (1^st^ L)
**Luspatercept**	ActRIIA ligand trapping	**phase 3** : luspatercept vs placebo (INDEPENDENCE)	anemia on JAK inh
KER-050	modified ActRIIA ligand trapping	phase 2 : monotherapy or + ruxolitinib	anemia on JAK inh/ineligible JAK inh
Nivolumab	PD-L1 inhibitor	phase 2 : nivolumab + fedratinib	int2/high risk R/R/suboptimal resp JAK inh (2^nd^ L)
Tagraxofusp	IL3 fused to diphteria targeting CD123 + LSC	phase 1	post-transplant maintenance

**Table:** **B) Ongoing clinical trials; active but not recruiting patients**

drug	mechanism of action	ongoing clinical trial active not recruiting	MF patients
Mivebresib	BET inhibitor	Phase 1b : monotherapy or + ruxolitinib or + navitoclax	≥ int1 risk R/R/intolerant JAK inh (2^nd^ L)
**Navitoclax**	BCL2, BCL-xL,BCL-W inhibitor	**phase 3** : navitoclax + ruxolitinib vs ruxolitinib (TRANSFORM-1)	int2/high risk JAK inh naive (1^st^ L)
Navitoclax	BCL2, BCL-xL,BCL-W inhibitor	phase 2 : monotherapy or + ruxolitinib (REFINE)	int2/high risk R/R/intolerant JAK inh (2^nd^ L)
Siremadlin Crizanlizumab Sabatolimab Rineterkib NIS793	MDM2 inhibitor Monoclonal antibody P-selectin Monoclonal antibody TIM-3 ERK inhibitor Monoclonal antiboby TGFβ1-2	phase 1b/2 : Platform Study of Novel Ruxolitinib Combinations in Myelofibrosis Patients (ADORE)	≥ int1 risk treated with ruxolitinib since 12 weeks (2^nd^ L)

**Abbreviations** : BID: twice a day; P/C: per physician choice; Int1: intermediate-1 risk following the DIPSS score; 1^st^ L: first line; JAK inh: JAK inhibitor; Int2: intermediate-2 risk following the DIPSS score; high risk: high risk following the DIPSS score; prePMF: prefibrotic primary myelofibrosis; 2^nd^ L: second line; vs: versus; BAT: Best available therapy; R/R relapsed/refractory; resp: response.

## JAK2 inhibitors

**Figure 1.**
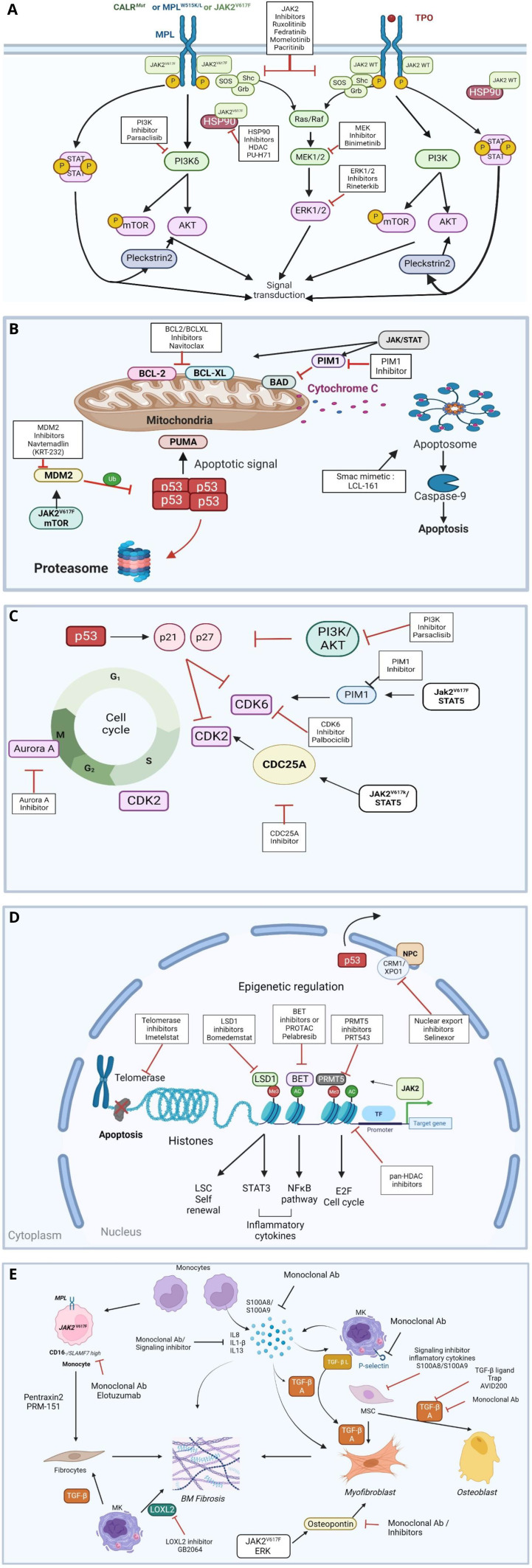
Targets and pathways of new therapies alone or in combination with JAK2 inhibitor (derived from reference ^43^). **A**) Signaling pathways, **B**) Apoptosis, **C**) Cell cycle, **D**) Epigenetic regulation, **E**) Myelofibrosis. The Figures have been created with bioRender.

Ruxolitinib was the first JAK inhibitor to be approved in the treatment of intermediate and high-risk MF. Ruxolitinib is a JAK1/2 inhibitor without selective effect on JAK2V617F or on oncogenic activation of JAK2WT by CALR or MPL mutants (see Figure 1A). It has become the reference for treatment of MF by reducing general symptoms, splenomegaly and improving the quality of life in around 50% of cases. The main toxicities are anemia and thrombocytopenia, in line with on-target effects. Despite an initial response, around 50% of the patients will discontinue ruxolitinib within 3 years mainly due to a lack/loss of response, cytopenia and/or progression to blast phase. However, it may also exert non-hematological side effects, the most prevalent being weight gain with increased levels of cholesterol and triglycerides and infections such as urinary infections and reactivation of herpes Zoster, tuberculosis and hepatitis B. There is some suspicion of an increased frequency of non-melanoma-skin cancers and B cell non-Hodgkin lymphoma. An abrupt termination may lead to the ruxolitinib discontinuation syndrome with a rapid increase in the spleen volume and a cytokine release syndrome^46^. Overall, in most patients, ruxolitinib has no significant effect on the progression of the disease and minor effect on the clonal disorder^4^. Some mutations such as *ASXL1* are predictive of a poor response to ruxolitinib. *ASXL1,* as well as *EZH2* and *RAS,* mutations can be acquired during treatment^47^. Ruxolitinib has a moderate effect on patient survival, except in patients with a prolonged response to treatment. For these reasons, other JAK2 inhibitors have been developed.

Fedratinib, a JAK2/FLT3 inhibitor, is the second approved JAK inhibitor in MF. Results are similar to ruxolitinib, with better splenomegaly response, but an increased non-hematological toxicity^48^. Fedratinib is indicated for patients intolerant or resistant to ruxolitinib, although in the US it can be used as a frontline therapy. Fedratinib has an increased gastrointestinal toxicity in comparison to ruxolitinib. There may be an increased risk of Wernicke encephalopathy due to thiamine deficiency as a consequence of a gastrointestinal toxicity and a controversial inhibition of the thiamine transporter-2. It is recommended to follow the thiamine levels during therapy^49^.

Pacritinib is a JAK2/FLT3 inhibitor and also inhibits IRAK1, implicated in myddosome, which regulates the synthesis of numerous pro-inflammatory cytokines including type I IFN. S100A8/9 (calprotectin) and IL33 that induce myddosome signaling are involved at the pre-fibrosis stage by reprogramming MSC into myofibroblasts^22,50^. However, no clear evidence exists that pacritinib inhibits marrow fibrosis development. Pacritinib also targets the activin receptor-type 1 (ACVR1/ALK2). The best indication of pacritinib concerns patients with marked thrombocytopenia and/or anemia. The main specific side effect is gastrointestinal toxicity, as described for fedratinib. In addition, in the initial clinical trial, it was suggested that pacritinib was associated with an increased risk of severe bleeding and cardiac events. It was not confirmed in the recent clinical trials^51^.

Momelotinib, recently FDA approved, is a JAK1/2 inhibitor that also targets ACVR1/ALK2 that transduces the BMP signal and regulates the liver synthesis of hepcidin^43,52^. Hepcidin is involved in the anemia of inflammatory disorders by sequestering the iron. Therefore, the principal advantage of momelotinib is to alleviate anemia^4^. The main specific side effects are thrombocytopenia, gastrointestinal toxicity, headache, peripheral sensory neuropathy and first-dose effect (dizziness, hypotension, or flushing).

In clinical trials, momelotinib and pacritinib exhibit similar effects as ruxolitinib on general symptoms and splenomegaly, but may correct the anemia in patients, alleviating the need for RBC transfusion^4^. Pacritinib seems to be a safe option for MF patients with severe thrombocytopenia^53^. It has to be confirmed by the undergoing randomized phase III study (PACIFICA).

None of these inhibitors display a selective inhibition on the oncogenic activation of JAK2, thus they do not induce molecular remission, whereas a strong JAK2 inhibition induces profound cytopenia. In agreement, resistance is not associated with JAK2 mutations in patients. When mutations in the RAS pathway occur during therapy, they can be present either in the *JAK2*V617F clone or *JAK2*WT cells^54^.

Therefore, there is a need for the development of JAK2V617F selective inhibitors or molecules targeting the activation of JAK2WT by CALR and MPL mutants (see last section).

## IFNα

**Figure 2.**
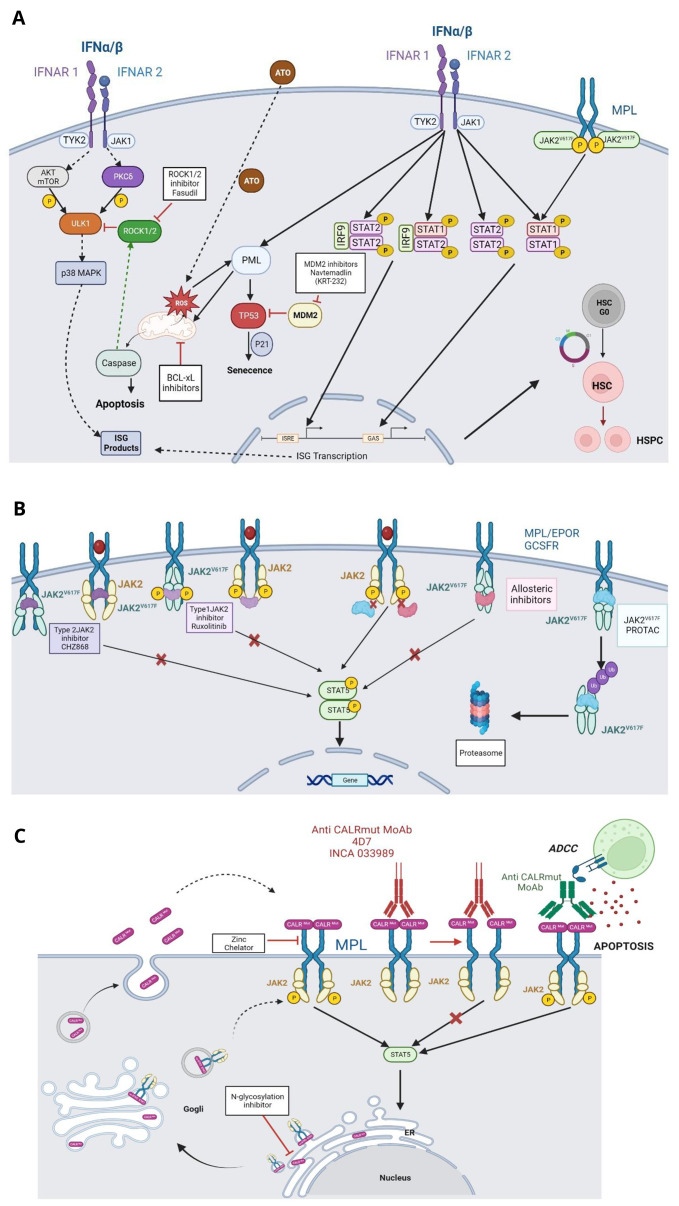
Approaches selectively targeting the disease drivers. **A**) Interferon alpha, **B**) JAK2V617F, **C**) Calreticulin mutants. The Figures have been created with bioRender.

IFNα is an old therapy of MPN, as it has been used since 1988 in ET and PV^55,56^. IFNα has become one of the major therapies in ET and PV for two reasons: (a) the toxicity of IFNα has been alleviated by using prolonged half-life type 1 IFN, and (b) it is the only available treatment that can act on the clonal disorder and may induce a deep molecular remission (in 25% of the cases)^57^. The efficacy of IFNα on the clonal disorder is dependent on the dose and the type of disease driver mutation. It is decreasingly efficient in targeting homozygous *JAK2*V617F, heterozygous *JAK2*V617F, *CALR* type-2 and *CALR* type-1^58^. However, the hematological remission is not dependent on the disease driver mutation. The role of associated mutations in the resistance to IFNα therapy is controversial. It clearly selects *DNMT3A* mutations whether they are biclonal or associated with *JAK2*V617F^59^. The mechanism by which IFNα therapy acts on the clonal disorder is not completely elucidated. There is strong evidence using mouse models that it targets *JAK2*V617F HSCs by inducing: (a) their entry in the cell cycle as normal HSCs, but without reentry in quiescence, thus principally leading to exhaustion of mutated cells (b) their apoptosis and eventually senescence through ROS accumulation and p53 induction, and (c) a shift of *JAK2*V617F HSCs to MK/myeloid biased HSCs with lower long-term reconstitution capacities^57,60,61^. (Figure 2A)

It is suggested that IFNα targets *JAK2*V617F HSCs, and to a much lower extent *CALR*mut HSCs, because JAK2V617F primes HSCs to an IFNα response by increasing STAT1 levels and activation, and consequently inducing expression of interferon stimulated genes (ISG)^62^. The effects of IFNα appear dependent of p53 and, although not demonstrated clinically, one has to exert caution with respect to the use of IFNα therapy in the presence of a *TP53* mutation. In addition, IFNα may induce an immune response against the malignant cells. Finally, it has been shown that the induction of the p38 pathway by IFNα through PKCδ and ULK1 is involved in the therapeutic effect of IFNα^63^.

At present, the use of an IFNα therapy in MF is limited by its toxicity with, as seen in its first trials, a low effect on the splenomegaly. However, preliminary results suggest that pegylated IFN may be efficient in MF, even in high-risk MF. As in PV, the treatment may decrease the *JAK2*V617F VAF and seems to have an effect on the survival in responder patients. It remains that treatment was stopped in nearly 75% of the patients due either to disease progression or intolerance^64^. Thus, it will be important to improve the IFN therapy to better target LSCs and to limit treatment length.

Combination of IFNα with ruxolitinib is being tested in clinical assays, although ruxolitinib as a JAK1/2 inhibitor decreases IFNα signaling^65^. However, ruxolitinib by its powerful anti-inflammatory effect may increase the tolerance to IFNα. Preliminary results suggest some efficacy of this combination in certain PV and MF patients, even those previously intolerant to IFNα therapy, with a decrease in *JAK2*V617F VAF and in fibrosis^65,66^. A recent preclinical study in *Jak2*V617F knock-in mice suggested that the use of fedratinib, a JAK2 inhibitor seems more efficient and may allow a decrease in the IFNα dose, and thus to have less toxic effects^67^.

Four other combinations have been experimentally efficient in preclinical studies (see Figure 2A):

- Arsenic trioxide with IFNα more efficiently targets the *JAK2*V617F LSC than IFNα alone, allowing long-term remission^68^. The combination increases *PML* overexpression and enhances PML nuclear bodies activity through sumoylation/oligomerization, leading to senescence^68^.
- A ROCK inhibitor (fasudil) enhanced the effects of IFNα on *JAK2*V617F hematopoietic cells in a mouse model^63^.Activation of ROCK1/2 acts as a feedback regulator of IFNα signaling by interacting with UKL1.
- Induction of p53 plays an important role in targeting *JAK2*V617F clone by IFNα. Combination of IFNα with a MDM2 inhibitor decreased the transplantability of human *JAK2*V617F HSPC into immunodeficient mice^69^. However, no clear synergy between IFNα and a MDM2 inhibitor (idasanutlin) was found in a phase 1 clinical trial in PV^70^.
- Combination of IFNα and 5-azacytidine seems extremely efficient in preclinical models^71^.

Overall, the risk of all these combinations is to increase the hematological toxicity of IFNα.

## Molecules in clinical development

### Telomerase inhibitor (Figure 1D)

Both telomere shortening and reactivation were observed in MPN. A molecule called imetelstat is a 13-mer lipid-conjugated oligonucleotide that inhibits telomerase activity. After initial testing in ET, imetelstat was tested in MF with a moderate effect on symptoms and spleen volume reduction, but apparently increased survival^43,72^. A phase 3 clinical trial is ongoing in MF with the goal to demonstrate an effect on overall survival^73^. Liver toxicity, although transient, is a limitation, as well as cytopenias. Thus, imetelstat could be an alternative for patients that are intolerant or resistant to JAK inhibitors and could represent one of the rare treatments that may induce disease modification.

Most other treatments are developed, essentially, in combination with ruxolitinib.

### Epigenetic regulator inhibitors (Figure 1D)

***BET inhibitors***

The Bromodomain and Extra-Terminal motif (BET) proteins interact with acetylated histones and transcription factors to induce gene expression such as *MYC* and genes downstream, NF-κB and TGF-β. Either small molecule inhibitors or BET degraders have been developed. In mouse models, the association of JQ-1 BET inhibitor and ruxolitinib was synergic in reducing fibrosis and the clonal disorder^74^. The effects are due to inhibiting NFκB transactivation and thus inflammation. In addition, a BET degrader targets the LSC in xenografts^75^.

Clinical trials are on-going using another BET inhibitor (pelabresib) alone or associated with ruxolitinib. Preliminary results suggest an efficiency with a decrease in *JAK2*V617F VAF, an effect on the marrow fibrosis and on anemia^76^.

***LSD1 inhibitor***

LSD1 (KDM1A), an H3K4 demethylase, is involved in transcriptional repression by the coREST complex^77^. It associates with GFI1/GFI1b and plays an important role in LSC self-renewal and differentiation. In addition, it regulates STAT3 activity. In *JAK2*V617F mouse models, LSD1 inhibition alleviates the myeloproliferative disorder and reduces MF acting in synergy with ruxolitinib^78^. It reduces the inflammation by decreasing inflammatory cytokine synthesis including IL8.

Bomedemstat, a LSD1 inhibitor, has been tested in human with some effects on the spleen volume, general symptoms, and the VAF of some mutations, such as *ASXL1* and fibrosis^77^.

***Protein arginine N-methyltransferase 5 (PRMT5)***

PRMT5 is a protein arginine-methylase, which methylates histone and non-histone proteins. PRMT5 is phosphorylated by JAK2V617F, impairing its methylase activity, which leads to HSC expansion and to increased erythroid differentiation^79^. However, it was subsequently shown that in MPN, PRMT5 is overexpressed, which positively regulates E2F1 target genes. In MPN mouse models, a PRMT5 inhibitor (C220) decreases myeloproliferation and systemic inflammation. Combination with ruxolitinib was more effective than individual therapy^80^. The PRMT5 inhibitor, PRT543, is now being evaluated in MF patients^44^.

***HDAC inhibitors (Figure 1A and D)***

HDACs are a large family of proteins that can deacetylate histones leading to gene repression but also gene activation. They also deacetylate non-histone proteins such as HSP90. The rationale to develop HDAC inhibitors was that HDACs 1-3 play an important role in HSC biology and self-renewal and HDAC1/2 are involved in erythroid and MK differentiation^81^. In addition, HSP90 behaves as chaperone for JAK2V617F, their interaction requiring HSP90 deacetylation by HDAC6^82^. *In vitro* combinations of a pan-HDAC inhibitor and ruxolitinib were very effective^83^. However, clinical trials were quite disappointing in MF with regards to toxicity (neutropenia and thrombocytopenia)^84^. Specific inhibitors of HSP90/HDAC6 or HDAC11 involved in oncogenic JAK/STAT signaling could be more relevant^82,85^.

###  Inhibitors of signaling molecules (Figure 1A)

Numerous signaling pathways are induced by the oncogenic activation of JAK2. Ruxolitinib only partially blocks these signaling pathways, such as the PI3K/AKT/mTOR and ERK/MAPK pathways, especially in *CALR*mut MPN.

***Inhibitors of the PI3K pathway***

The PI3K pathway through AKT and mTOR plays an important role in both cell proliferation and survival for key targets p27 and BAD. Both *in vitro* studies and MPN mouse models have shown that pan-PI3K, mTOR, and AKT inhibitors have major effects on the MPN, with a synergistic effect with ruxolitinib^86–88^. However, pan-PI3K inhibitors have an important dose-dependent toxicity. PI3Kδ is the most expressed PI3K isoform in MF CD34^+^ cells. Parsaclisib, a new generation PI3Kδinhibitor, is being tested in phase 3 clinical trials of MF in association with ruxolitinib^4,43,60,89^. However, the clinical trial has been recently discontinued due to a lack of efficiency on the reduction of spleen volume. Pleckstrin-2 could be another target of this pathway^90^.

***MEK and ERK inhibitors (Figure 1 A and E)***

The RAS/MAPK pathway plays an important role in the resistance to ruxolitinib, with the frequent occurrence or selection of mutations on this pathway during ruxolitinib treatment^54^. Mouse models have shown that MEK/ERK inhibition increases the effects of JAK2 inhibition on the myeloproliferation, MF and systemic inflammation by decreasing the level of numerous pro-inflammatory cytokines and osteopontin^33,91^. The efficacy of a combination between an ERK inhibitor (rineterkib) and ruxolitinib is being tested in a clinical trial^43^.

***PIM (Figure 1 B and C)***

The PIM family is composed of serine-threonine kinases that are the direct transcriptional targets of STAT5. They are constitutively active kinases only regulated by their expression. They cooperate with MYC in oncogenesis. PIM1 is overexpressed in MF CD34^+^ cells. Genetic deletion or pharmacologic inhibition of PIM together with ruxolitinib has a major effect on the myeloproliferative disorder, reducing the fibrosis by decreasing the TGF-β1 level^92–94^.

***HSP90***

HSP90 is as a molecular chaperone with numerous substrate proteins, including JAK2V617F. Its inhibition leads to the degradation of total and phospho-JAK2. In MPN mouse models, the combination of ruxolitinib with an HSP90 inhibitor was more efficient than ruxolitinib alone by further decreasing JAK/STAT signaling^95,96^. An initial clinical trial with AUY922 induces a severe non-hematologic toxicity^97^. A combination of PU-H71 with ruxolitinib is currently being evaluated in MF patients.

### Apoptosis inducers (Figure 1C) and Cell cycle inhibitors (Figure 1D)

Targeting key molecules implicated in proliferation and cell survival downstream of JAK2 appears as an interesting therapeutic approach.

***CDK4/6 inhibitor***

CDK4/6 are key molecules in cell cycle entry as they phosphorylate Rb when activated. In contrast to CDK4, CDK6 is involved in stress hematopoiesis and hematological malignancies and regulates transcription in both kinase-dependent and -independent manners. CDK6 is overexpressed in CD34^+^ cells from MPN. *JAK2*V617F regulates CDK6 through CDC25A or PIM. Several CDK4/CDK6 inhibitors, including palbociclib, have been approved for the treatment of solid tumors, with an acceptable hematological toxicity. *CDK6* ablation has a major effect on the myeloproliferative disorder, but also on inflammation inhibiting NFκB and TGF-β signaling^98^. CDK4/CDK6 inhibitors exert a synergistic therapeutic effect with ruxolitinib and a PIM inhibitor in mouse models^92,99^. CDK6 PROTAC is under development^100^.

***Aurora A kinase inhibitor***

Aurora A is a serine threonine kinase involved in G2/M transition and the organization of the spindle, regulating many other targets. Aurora A inhibition in acute megakaryoblastic leukemia leads to differentiation^101^. In mouse models, its inhibition ameliorated MF^102^. A phase 1 clinical study was performed with a response in 30% of cases, including a decrease in MF and *JAK2*V617F/*CALR* VAF without major toxicity^103^.

***BCL2 family inhibitor***

The BCL2 family includes pro-survival molecules such as BCL2, BCL-xL and MCL1 and pro-apoptotic molecules. BCL2 and MCL1 regulate the survival of early stages of hematopoiesis whereas BCL-xL is indispensable for erythroblasts and MKs. BCL-xL and BCL2 are direct targets of STAT5 and indirect targets of the PI3K pathway and are overexpressed in MPN^60^. Venetoclax, a BCL2 inhibitor, is now a promising therapy in AML. In MPN, there is a need to also target BCL-xL. Navitoclax is an inhibitor of BCL2, BCL-xL and BCL-W. In *JAK2*V617F cell lines, there was a synergy between JAK2 inhibition and navitoclax, also leading to reversal of the ruxolitinib resistance^104^. Phase 2 clinical trials of this combination were promising with a very significant decrease in the spleen volume, a decrease of fibrosis, including some patients with a resolution of the marrow fibrosis and of the *JAK2*V617F*/CALR* VAF^44,105^. Furthermore, the thrombocytopenia was manageable and, surprisingly, the anemia was partially corrected. Phase 3 clinical trials are ongoing. This combination seems to be one of the most effective.

***MDM2 inhibitor and the p53 pathway***

MDM2 is an E3 ubiquitin ligase that negatively regulates p53 by degradation. p53 is a potent negative regulator of JAK2V617F signaling that is downregulated by MDM2 overexpression by JAK2V617F^106^. Results of a phase 2 trial with navtemadlin MDM2 inhibitor were encouraging, and further clinical trials including a phase 3 trial are being conducted in monotherapy or in combination with ruxolitinib in MF^107^. Some specific inhibitors of PPM1D, another P53 regulator, have been developed that could be combined with MDM2 inhibitors at lower doses to avoid gastro-toxicity^108^. However, the risk of such treatments is to select *TP53* mutated subclone, thus patients with a *TP53* mutation must be excluded^109^.

***Selective inhibitors of nuclear export (Figure 1D)***

A shRNA screening on a JAK2V617F cell line identified a particular sensitivity to the inhibition of the nuclear export transport (NE) machinery^110^. These results were confirmed in primary MF CD34^+^ cells and in a mouse model with a combination of a specific NE compound (selinexor) and ruxolitinib^110^. The effects of inhibiting NE are presumably related to the accumulation of tumor suppressor proteins in the nucleus, especially p53. A phase 2 clinical trial with selinexor is ongoing in MF JAK2 inhibitor intolerant patients^111^.

***Other inducers of apoptosis***

LCL-161 is a second mitochondrial activator of caspases (SMAC) mimetic that antagonizes inhibitors of apoptosis (IAP). In preclinical studies, LCL-161 induced *JAK2*V617F cell apoptosis that was rescued by JAK2 inhibition^112^. Thus a phase-2 clinical trial in monotherapy was performed and gave encouraging results in old high-risk patients^113^.

### Targeting the marrow fibrosis (Figure 1E)

These approaches are based on the marrow fibrosis mechanism.

***First Approach***

The first approach targets the external cues involved in fibrosis development or their signaling.

- A first trial was performed using an anti-TGF-β1antibody, but it only includes 3 patients with a response essentially on the anemia scale^114^. A second assay was conducted with AVID 200, a TGF-β1/3 ligand trap (TGF-β receptor ectodomains fused to a human Fc domain). Treatment of GATA1^low^ mice by AVID200 reduced marrow fibrosis^115^. A preliminary clinical trial led to the improvement of thrombocytopenia. Another trial is programmed with a new anti-TGF-β1 monoclonal antibody (MoAb).
- Approaches blocking inflammatory cytokines or their signaling such as IL8, IL13 or IL1β and S100A8/S100A9 were promising in MPN mouse models with synergistic or additive effects with ruxolitinib^28,31,32,116^. Interestingly, pacritinib by inhibiting IRAK1 may inhibit the signaling of both IL1β and S100A8/S100A9^22^.
- JAK2V617F decreases the number of nestin cells involved in HSC regulation by inducing their apoptosis, due to an IL1β-induced damage of sympathetic nerves that innerve nestin cells^117^. Mirabegron, a β-3 sympathomimetic agonist, has been tested in a phase 2 clinical trial leading to a slight increase in nestin cells and decrease in fibrosis, but without altering the *JAK2V617F* VAF^118^.

***Second Approach***

- Serum amyloid P (pentraxin 2) is capable of suppressing fibrosis of many organs by inhibiting the differentiation of monocytes into fibrocytes and by acting on macrophages and neutrophils. The recombinant form, PRM-151, has been shown to inhibit *in vitro* fibrocyte differentiation from PMF^36^. In addition, it prolonged survival of mice xenotransplanted with PMF hematopoietic cells. Therefore, PRMT-151 has been tested in a phase 2 clinical trial with a regression of fibrosis and improvement of cytopenia in some patients^119^. It has been recently also tested in association with ruxolitinib in phase 1 and 2 clinical trials with an acceptable toxicity^120^.
- Another closely related approach consists of the use of a SLAMF7 antibody to prevent differentiation of monocytes into fibrocytes^37^.

***Third Approach***

This approach concerns the targeting of Lysyl oxidase-like-2 (LOXL2) and P-selectin.

- LOXL2 is an enzyme that stabilizes the ECM by crosslinking collagen that is also involved in MK expansion induced by PDGFB^24^. While it is overexpressed by MKs in marrow fibrosis mouse models, its pharmacological inhibition reduces marrow fibrosis^121^. A phase 2 clinical trial using an anti-LOXL2 MoAb was conducted in MF patients alone or in association with ruxolitinib, the results being disappointing^122^. A phase 2 clinical trial is ongoing using an inhibitor of LOXL2, GB2064. In 4 patients a reduction in collagen fibrosis was observed at 6 months^123^.
- P-selectin is a glycoprotein that is present in platelet and MK α-granules. It is translocated to the cell surface after activation. P-selectin is involved in the emperipolesis of granulocyte precursors by MKs, leading to TGF-β1 release^35^. In the GATA1^low^ mouse model, *SELP* (P-selectin gene) ablation or P-selectin blockage by a MoAb impairs MF development^124^. A phase 1 trial is programmed using a combination of ruxolitinib and a MoAb^44^.

### Targeting cytopenia

All the previous approaches aim also to correct the cytopenia by modifying the disease. In addition, specific therapies for anemia are being tested in MF beyond the classical therapies (androgens, IMID agents and ESA)^4,125^. The first consists of a ligand trap strategy using the extracellular domain of ACVRIIB and IIA fused to the human IgG Fc domain for luspatercept and sotatercept, respectively. These ligand traps bind some members of the TGFβ superfamily, such as GDF11 that negatively regulates late stages of erythroid differentiation through SMAD2/3^126^. In addition, they may decrease hepcidin synthesis by trapping some BMPs involved in its synthesis and by increasing the level of erythroferrone as a consequence of an increased erythroid maturation and thus they mobilize the iron store. Both ligand traps have been tested in phase 2 clinical trials alone or in association with ruxolitinib to correct the anemia of MF. Around one third of the patients had a clinical response^43,125^. In addition, such therapies may have an effect on cachexia of advanced MF patients.

The other approaches aim to target hepcidin, which is regulated at the transcriptional level by IL6 (and other members of the family) through the GP130/JAK2/1/STAT3 pathway and by BMPs1/6, the first pathway being regulated by inflammation, the second by an iron overload. Momelotinib targets both pathways of hepcidin regulation^52^. In addition, an ACVR1/ALK2 inhibitor (BMP pathway) is being tested in combination with ruxolitinib in anemia of MF^43,125^.

In the future, other approaches could be used in MF, including hepcidin antagonists, whereas hepcidin mimetics appear quite efficient in the therapy of PV erythrocytosis. 

### New approaches specifically targeting the clonal disease

Presently, most new therapies are based on the association of ruxolitinib with other molecules. However, the risk of drug combination is increased toxicity. It is important to develop new approaches targeting either specifically the disease driver mutants (JAK2V617F, CALR or MPL mutants) or directly the LSC.

These last years, there has been some progress in the development of an immunotherapy against *CALR*mut and *JAK2*V617F MPNs.

***Immunotherapy***

*JAK2*V617F and *CALRmut* MPN clones may escape T cell surveillance, even if CD4 or CD8 T cells directed against these mutants exist. These immune cells are rendered non-functional by the PD1/PD-L1 axis. However, a phase 2 clinical trial using pembrolizumab, an anti-PD1 antibody, failed to induce a clinical response^127^.

*CALR*mut generate a new C-terminus, with neo-antigens that could be targeted by immunotherapy. However, this approach may be limited by several factors: (a) MHC-1 having a high affinity for these neo-epitopes is under-represented in MPN patients^128^; (b) huge levels of soluble mutated CALR are present that inhibit the phagocytosis of dying cancer cells by dendritic cells and suppress the effects of PD-1 blockade^129^; and (c) CALR is implicated in the peptide loading on MHC-1^130^. A first trial using a peptide vaccine showed no clinical response, although a strong immune response was observed mainly involving CD4^+ ^T cells^131^. Trials are ongoing using a vaccine associated with an immune checkpoint inhibitor. Alternatively, it has been shown that a heteroclitic peptide with an optimized presentation by MHC-1 may permit to overcome this immunosuppressive state^128^.

***LSC targeting***

In MF, LSCs are skewed towards the MK lineage and aberrantly express MK/platelet antigens, such as G6B, defining new targets for immunotherapy^132^. In addition, CD123, the IL3α-receptor, is expressed on LSCs of numerous myeloid malignancies. Presently, tagraxofusp, a fusion protein consisting of IL3 fused to diphteria toxin has been tested in a phase 1/2 clinical trial, but with modest results^133^.

***Direct targeting of the mutated disease drivers (Figure 2B and C)***

Unexpectedly, CALRmut can be targeted more easily than JAK2V617F.

Using a large screening approach, it has been shown that inhibition of N-glycosylation by several molecules can target the oncogenic CALRmut signaling by inhibiting the MPL membrane expression^134^. Such an approach could be extended to MPL mutants. In addition, CALRmut multimerization is dependent on zinc, thus zinc chelators could be a valuable approach^135^.

The development of MoAbs targeting specifically the CALRmut induced positive expectations. Anti-CALRmut antibodies may either impair abnormal signaling of MPL/JAK2 complexes or induce an immune reaction. Three different anti-CALRmut MoAbs, namely B3, 4D7 and INCA033989, were generated^136–138^. 4D7 and INCA033989 disrupt CALR mutant/MPL signaling^136,137^. These antibodies inhibit *in vitro* the growth of CALRmut/MPL cell lines and patient cells including in xenografts. In addition, this approach appears to have a major effect on the disease development in a murine model by targeting the LSC^137,138^. A clinical trial is in progress with INCA033989. Another antibody against CALRmut injected in *CALR*del52 mice model led to a very rapid normalization of platelet count and a decrease of LSK^139^. This response may reflect immunodepletion rather than signaling interruption. Finally, a synthetic peptide inhibiting the interaction between MPL and CALRmut was able to inhibit the constitutive MPL/JAK2 signaling *in vitro*^140^.

Concerning JAK2, due to the frequently acquired resistance to type I JAK inhibitors (ruxolitinib, fedratinib), two type II JAK inhibitors that interact with a JAK2-inactive conformation were efficiently tested in preclinical studies, but were toxic^141^. A new type II JAK2 inhibitor (*AJ1*-*10502*) showed improved efficacy in comparison to ruxolitinib, with a selective effect on *JAK2*V617F cells^142^.

Specific JAK2V617F allosteric inhibitors theoretically seem the more straightforward approach to directly impact the clone without inducing cytopenia. Numerous progresses have been obtained in the structure of JAK2V617F by mutational approaches and by ultrastructural analysis^143–145^. Recently, a cryo-EM structure of the complex between IFNAR2/JAK1V657F, the homologous mutation of JAK2V617F, has been obtained^146^. Despite the limitation using JAK1, this new structure indicated dimerization of the mutated PK domains of JAK1. New small molecules inhibiting the dimerization of JAK2V617F may be promising. A limitation of this approach is that the conformation of JAK2V617F is close to the conformation of JAK2WT associated with the IFNγ receptor after ligand binding, raising the risk that such molecules may thus inhibit IFNγ signaling^144^.

Another approach will be to target JAK2V617F degradation. This has been recently reported in ALL by determining the structure of the interaction of ruxolitinib and baricitinib with JAK2^147^. A better knowledge of the JAK2V617F structure may also allow the development of specific PROTAC.

A last approach is to target the conformational differences between interaction of JAK2V617F or JAK2WT interacting with MPL and EPOR. This strategy using diabodies has been successful in targeting the EPOR/JAK2V617F interaction^148^.

## In conclusion

Presently, efforts are geared towards finding new therapies that would profoundly modify the disease either based on drug combination or on the development of new compounds, some directly targeting the disease drivers. All these efforts would likely lead in the future to major advances in the treatment of MF. 
